# [Retracted] Arctigenin, a natural lignan compound, induces G_0_/G_1_ cell cycle arrest and apoptosis in human glioma cells

**DOI:** 10.3892/ol.2023.14173

**Published:** 2023-12-05

**Authors:** Aisha Maimaitili, Zunhua Shu, Xiaojiang Cheng, Kadeer Kaheerman, Alifu Sikandeer, Weimin Li

Oncol Lett 13: 1007–1013, 2007; DOI: 10.3892/ol.2016.5474

Following the publication of this paper, a concerned reader drew to the attention of the Editorial Office that, for the flow cytometric plots shown in Fig. 4A on p. 1012, in comparing the dats shown for the T98G and U87MG experiments, the plots in the lower pair of quadrants looked remarkably similar, whereas the data in the upper pair of quadrants were clearly different. Upon assessing the matter raised by the reader, the Editor of *Oncology Letters* has determined that this paper should be retracted from the journal on account of a lack of confidence in the presented data. The authors were asked for an explanation to account for these concerns, but the Editorial Office did not receive a reply. The Editor apologizes to the readership for any inconvenience caused.

